# Structural basis of polyethylene glycol recognition by antibody

**DOI:** 10.1186/s12929-019-0589-7

**Published:** 2020-01-07

**Authors:** Cheng-Chung Lee, Yu-Cheng Su, Tzu-Ping Ko, Li-Ling Lin, Chih-Ya Yang, Stanley Shi-Chung Chang, Steve R. Roffler, Andrew H.-J. Wang

**Affiliations:** 1 Institute of Biological Chemistry, Academia Sinica, Taipei, Taiwan; 2 Department of Biological Science and Technology, National Chiao Tung University, Hsin-Chu, Taiwan; 3 Medigen Biotechnology Corporation, Taipei, Taiwan; 4 Institute of Biotechnology, National Taiwan University, Taipei, Taiwan; 5 Institute of Biomedical Sciences, Academia Sinica, Taipei, Taiwan

**Keywords:** X-ray crystallography, PEG-fab complex, Dimer formation, Protein-protein interaction, Crown ether

## Abstract

**Background:**

Polyethylene glycol (PEG) is widely used in industry and medicine. Anti-PEG antibodies have been developed for characterizing PEGylated drugs and other applications. However, the underlying mechanism for specific PEG binding has not been elucidated.

**Methods:**

The Fab of two cognate anti-PEG antibodies 3.3 and 2B5 were each crystallized in complex with PEG, and their structures were determined by X-ray diffraction. The PEG-Fab interactions in these two crystals were analyzed and compared with those in a PEG-containing crystal of an unrelated anti-hemagglutinin 32D6-Fab. The PEG-binding stoichiometry was examined by using analytical ultracentrifuge (AUC).

**Results:**

A common PEG-binding mode to 3.3 and 2B5 is seen with an S-shaped core PEG fragment bound to two dyad-related Fab molecules. A nearby satellite binding site may accommodate parts of a longer PEG molecule. The core PEG fragment mainly interacts with the heavy-chain residues D31, W33, L102, Y103 and Y104, making extensive contacts with the aromatic side chains. At the center of each half-circle of the S-shaped PEG, a water molecule makes alternating hydrogen bonds to the ether oxygen atoms, in a similar configuration to that of a crown ether-bound lysine. Each satellite fragment is clamped between two arginine residues, R52 from the heavy chain and R29 from the light chain, and also interacts with several aromatic side chains. In contrast, the non-specifically bound PEG fragments in the 32D6-Fab crystal are located in the elbow region or at lattice contacts. The AUC data suggest that 3.3-Fab exists as a monomer in PEG-free solution but forms a dimer in the presence of PEG-550-MME, which is about the size of the S-shaped core PEG fragment.

**Conclusions:**

The differing amino acids in 3.3 and 2B5 are not involved in PEG binding but engaged in dimer formation. In particular, the light-chain residue K53 of 2B5-Fab makes significant contacts with the other Fab in a dimer, whereas the corresponding N53 of 3.3-Fab does not. This difference in the protein-protein interaction between two Fab molecules in a dimer may explain the temperature dependence of 2B5 in PEG binding, as well as its inhibition by crown ether.

## Background

Polyethylene glycol (PEG) is a water-soluble, low immunogenic and biocompatible polymer formed by repeating units of ethylene glycol [1]. A wide range of therapeutic compounds have been modified by PEG to improve their solubility, circulation time and bioavailability for medical use [2–5]. PEGylation enlarges the size of the conjugated compounds and hinders enzymatic digestion, thereby increasing their half-life in circulation to exert long-term therapeutic effects [1]. To successfully develop PEGylated medicines, a simple and sensitive method to detect PEG for pharmacokinetic studies is strongly desired. PEG is the common component of PEGylated compounds including peptides, proteins, nucleic acids, small molecule drugs and nanoparticles. In this regard, we have developed monoclonal antibodies that can bind specifically to PEG for universal detection and quantification of PEGylated drugs by anti-PEG sandwich enzyme-linked immunosorbent assay (ELISA) [6, 7]. The anti-PEG antibodies have been further engineered for cancer-targeting therapy to universally deliver PEGylated nanoparticles to tumors by using bispecific PEG-binding antibodies that simultaneously bind to PEG on the nanomedicines and to membrane receptors (e.g., epidermal growth factor receptor, or EGFR) on the cancer cells [8–10]. Because the underlying mechanism for specific PEG binding has not been elucidated, by solving complex crystal structures of the anti-PEG Fab with PEG and by analyzing their interactions, we provide a possible basis for designing improved anti-PEG antibodies with higher affinity.

Previously, by immunization of mice with a PEG conjugate we first generated an anti-PEG monoclonal antibody (mAb) AGP3/IgM [11]. Later another anti-PEG mAb E11/IgG was also obtained [12]. Further identification of the second-generation mAbs AGP4/IgM and 3.3/IgG allowed more sensitive detection of a broader range of PEGylated compounds [7]. Recently, by somatic hyper-mutation, the variant mAb 2B5/IgG obtained from the parental mAb 3.3/IgG displayed greater affinity for PEG at 4 °C, but the affinity was decreased at higher temperatures (Additional file 1: Table S1) [13]. 2B5 differs from 3.3 in three amino acid residues (Additional file 1: Table S2), including V23 in the heavy chain and K53 and P55 in the light chain, which correspond to A23(H), N53(L) and A55(L) in 3.3. (H and L in parentheses denote residues in the heavy chain and light chain, respectively.) The temperature-selective binding of 2B5 was attributed to K53(L) because it was abolished by mutating K53(L) back to asparagine [13]. In this study, the Fab fragments of mAb 3.3 and 2B5 were each co-crystallized with PEG and both structures were determined by X-ray diffraction. The structures revealed a common mode of specific PEG binding. It differs entirely from that in the PEG-containing crystal of a human 32D6-Fab against influenza hemagglutinin [14], whose structure was determined in this study also.

## Material and methods

### Production of the 3.3 and 2B5 antibodies

The development of antibodies 3.3 and 2B5 have been described previously [7, 13]. 2.5 × 10^7^ of 3.3 or 2B5 hybridoma cells in 15 mL culture medium (DMEM, 5% FBS) were inoculated into a CELLine CL 1000 two-compartment bioreactor (INTEGRA Biosciences AG). The antibody-containing culture medium was harvested every 7 days and then purified by protein A Sepharose 4 Fast Flow chromatography (GE Healthcare). The collected antibody solution was dialyzed against PBS and sterile filtered. Antibody concentrations were determined by the bicinchoninic acid (BCA) protein assay (Thermo Scientific).

### 3.3-fab and 2B5-fab fragmentation

Papain solution (0.1 mg/mL) (Sigma-Aldrich) was prepared in PBS supplemented with 20 mM L-cysteine and 20 mM EDTA (Sigma-Aldrich) and the pH was then adjusted to 7.2. An equal volume of purified 3.3 or 2B5 anti-PEG antibody (2 mg/mL) was added to the papain solution and incubated at 37 °C for 2.5 h. One-tenth volume of 0.3 M iodoacetamide solution (Sigma-Aldrich) was added to stop the reaction. The 3.3 and 2B5 anti-PEG Fab fragments were purified by affinity chromatography on a PEG affinity column, generated by swelling 1 g of CNBr-activated Sepharose 4B (GE Healthcare) in 1 mM HCl (pH 3) for 30 min, washing with coupling buffer (0.1 M NaHCO_3_, pH 8.3) and adding 5 moles of methoxy-PEG_30K_-amine (Laysan Bio) per mL gel in coupling buffer for 4 h at 25 °C. Remaining active groups on the CNBr-activated Sepharose were blocked by adding 1/10 volume of 1 M Tris (pH 8) to the gel at 25 °C for 2 h. The PEG coupled Sepharose was washed with 0.1 M acetate buffer (pH 4) containing 0.5 M NaCl followed by 0.1 M Tris (pH 8) containing 0.5 M NaCl. Papain digested antibodies were loaded to the PEG-resin column at 4 °C for 45 min and washed with cold PBS to remove papain and Fc fragments. The PEG-resin bound anti-PEG Fab fragments were eluted with 100 mM glycine buffer (pH 3) and dialyzed against 20 mM Tris buffer (pH 7.5).

### Recombinant protein preparation of 32D6-fab

The 32D6-Fab expression vector was derived from the IgG expression plasmid, pIgG (U.S. patent No. 5736137), which did not contain the C_H_2 and C_H_3 domains of the heavy chain but contained an additional His-tag at the C-terminus of the C_H_1 domain of the heavy chain [14]. The 32D6-Fab was expressed in Expi293F cells and purified via a HisTrap excel column using an imidazole gradient from 10 mM to 500 mM in a buffer solution of 20 mM sodium phosphate, 500 mM NaCl, pH 7.4. The fractions containing 32D6-Fab were eluted at 300 mM imidazole.

### Crystallization and data collection

The complex crystals of the 3.3-Fab/PEG and 2B5-Fab/PEG/CR were grown by mixing 1 μL protein solution (10 mg/mL) with 1 μL reservoir solution using the sitting-drop vapor diffusion method at 18 °C and 10 °C, respectively. The 3.3-Fab/PEG crystals were obtained in a reservoir solution of 18% (*w/v*) PEG-4000, 10% (*v/v*) 2-propanol, 1% (*w/v*) PEG-2000-MME, 0.1 M Na-citrate, pH 5.6. 2B5-Fab/PEG/CR crystals were grown in the reservoir solution of 50 mM 18-crown-6, 30% (*w/v*) PEG-8000, 0.2 M lithium sulfate, 0.1 M acetate, pH 4.5. Both crystals were flash-cooled with 20% glycerol (*v/v*) as a cryo-protectant for X-ray data collection at cryogenic temperatures. The diffraction data of 3.3-Fab/PEG crystals were collected on beamline BL12B2 of the SPring-8 synchrotron in Japan. The 2B5-Fab/PEG/CR data set was collected at National Synchrotron Radiation Research Center (NSRRC) beamline BL15A in Taiwan.

Crystals of 32D6-Fab/PEG were prepared by mixing 1 μL protein solution (10 mg/mL) with 1 μL reservoir solution and 0.5 μL additive solution using the sitting-drop vapor-diffusion method at 18 °C. The crystals were grown from a reservoir solution consisting of 42% v/v PEG-600, 0.1 M CAPSO, pH 9.6 with the additive solution of Silver Bullets No.96 which contains 0.16% (w/v) Aspartame, 0.16% (w/v) Gly-gly-gly, 0.16% (w/v) Leu-gly-gly, 0.16% (w/v) Pentaglycine, 0.16% (w/v) Tyr-ala, 0.16% (w/v) Tyr-phe, and 0.02 M HEPES sodium pH 6.8 (Hampton Research). The diffraction data of 32D6-Fab/PEG were collected at Taiwan Photon Source (TPS) beamline TPS-05A of NSRRC in Taiwan. All diffraction data were processed and scaled by using the program HKL-2000 [15].

### Structure determination and refinement

All Fab/PEG complex crystal structures were determined by molecular replacement using the program MOLREP of the CCP4 program suite [16], and the crystal structure of the E317 IgG_1_ Fab fragment (PDB: 3W9D) [17] was used as a search model for structure determination of the 3.3-Fab/PEG and 2B5-Fab/PEG/CR crystals, which belong to space group *P*2_1_ and *P*4_3_2_1_2, respectively. The crystal structure of 32D6-Fab/PEG was determined by using the Fab part of PDB entry 6A4K [14] as a search model. The 32D6-Fab/PEG crystals belong to space group *P*3_2_21.

The crystal structures were refined by using PHENIX [18]. Throughout the refinement, 5% of randomly selected data were set aside for cross validation with *R*_free_ values. Manual modifications of the models were performed by using the program COOT [19]. Difference Fourier (Fo-Fc) maps were calculated to locate the bound ligands and solvent molecules. Data collection and final model statistics are shown in Table 1. The molecular figures were produced with PyMOL [21] and Chimera [22]. The atomic coordinates and structure factors of 3.3-Fab/PEG, 2B5-Fab/PEG/CR and 32D6-Fab/PEG complexes have been deposited in the Protein Data Bank with accession codes 6JU0, 6JWC and 6JP7, respectively.

**Table 1:** Data collection and refinement statistics^a^

	3.3-Fab/PEG	2B5-Fab/PEG/CR	32D6-Fab/PEG
Data collection			
Wavelength (Å)	1.0	1.0	1.0
Space group	*P*2_1_	*P*4_3_2_1_2	*P*3_2_21
Unit-cell *a, b, c* (Å)	69.30, 177.35, 89.02	98.90, 98.90, 96.71	73.66, 73.66, 191.25
α, β, γ (°)	90.0, 92.0, 90.0	90.0, 90.0, 90.0	90.0, 90.0, 120.0
Resolution (Å)	25.0–2.6 (2.69–2.60)	20.0–2.3 (2.38–2.30)	30.0–1.91 (1.98–1.91)
Unique reflections	64,434 (6420)	21,952 (2123)	47,075 (4515)
*R*_pim_ (%)	5.2 (36.1)	3.0 (29.6)	4.2 (21.8)
Average *I*/σ(*I*)	15.1 (2.2)	25.3 (2.8)	16.5 (2.1)
Completeness	98.6 (98.5)	99.9 (100.0)	98.5 (96.4)
Redundancy	3.1 (3.0)	7.0 (7.0)	3.5 (3.2)
Average CC_1/2_	0.928 (0.699)	0.954 (0.808)	0.951 (0.854)
Z	4	1	1
Refinement			
No. of reflections	63,647 (5475)	21,890 (2094)	43,899 (2744)
*R*_work_ (%)	21.08 (29.71)	18.70 (24.89)	16.87 (20.53)
*R*_free_ (%)	24.01 (34.03)	22.55 (27.00)	21.35 (26.74)
No. of atoms/Avg. B factor (Å^2^)			
Protein	13,044/45.4	3258/37.0	3445/22.9
PEG + Crown ether	157/53.5	154/43.3	53/33.1
Water molecules	826/45.1	387/42.5	567/36.1
RMSD from ideal values			
Bond lengths (Å)	0.0024	0.0025	0.0076
Bond angles (°)	0.69	0.61	0.95
Ramachandran statistics (%)^b^			
Favored	98.09	97.37	97.11
Allowed	1.67	2.63	2.45
Outliers	0.24	0.00	0.44
Clash score	3.79	3.44	2.48
MolProbity score	1.53	1.26	1.26
PDB code	6JU0	6JWC	6JP7

^a^Values corresponding to the highest resolution shell are shown in parentheses

^b^The stereochemistry of the model was validated with MolProbity [20]

### Analytical ultracentrifugation (AUC)

The 3.3-Fab protein samples at two different concentrations, 0.1 mg/mL and 0.3 mg/mL, in 25 mM Tris-HCl buffer, with and without 0.1% PEG-550-MME were analyzed by AUC. Sedimentation velocity (SV) measurements were performed at 200 kg (50,000 rpm) by using a 4-hole AnTi60 rotor at 20 °C in a Beckman Optima XL-I AUC equipped with absorbance optics. Standard 12 mm aluminum double-sector centerpieces were filled with protein solution, and the reference cell contained the blank buffer. Quartz windows were used along with absorbance optics (OD_280_) in a continuous mode without averaging. No time interval was set between scans. Data were analyzed with a c(s) distribution of the Lamm equation solutions calculated by the program SEDFIT Version 12. The software Sednterp (http://www.jphilo.mailway.com) was used to estimate protein partial specific volume (Vbar), buffer density (0.99966 g/mL), and buffer viscosity (0.010167 P). The Vbar value of 3.3-Fab was 0.7300 mL/g.

## Results

### Fab/PEG complex structures

**Fig. 1:**
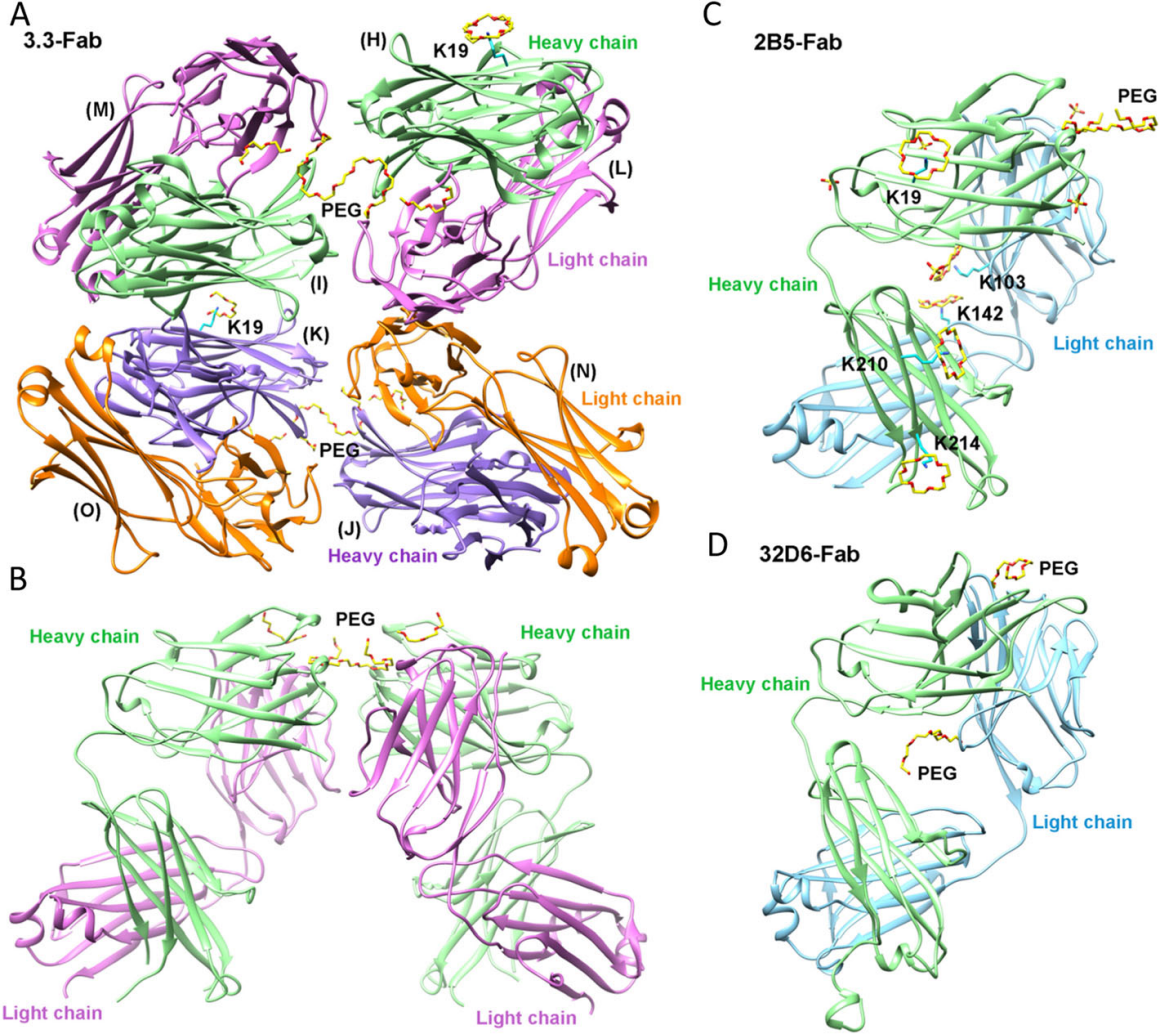
Crystal structures of Fab-PEG complex. The protein parts are shown as ribbons diagrams with different colors distinguishing the different chains in the crystal structures. **a** One asymmetric unit of the 3.3-Fab/PEG crystal contains four molecules of Fab. **b** A dimeric 3.3-Fab/PEG complex that comprises the protein chains H/L and I/M is rotated by about 90° from (**a**) and viewed sideways from the pseudo-dyad with the bound PEG on the top. **c**, **d** The asymmetric units of the 2B5-Fab/PEG/CR and 32D6-Fab/PEG complex crystals are shown in a similar orientation as in (**b**). Ligands including PEG, crown ether, and sulfate are shown as stick models

The monoclinic crystal of 3.3-Fab/PEG complex contains four Fab fragments in an asymmetric unit (Fig. 1a). Each Fab comprises the N-terminal V_H_ and C_H_1 domains of the heavy chain (named H, I, J, K) and the V_L_ and C_L_ domains of the light chain (L, M, N, O). The asymmetric unit can be divided into two pairs of Fab (H/L, I/M and J/N, K/O) related by a non-crystallographic two-fold symmetry. Each pair of Fab also contains a local pseudo-dyad axis (Fig. 1b). However, the latter pseudo-dyad axes do not coincide with one another. Because the X-ray diffraction data were collected to only 2.6-Å resolution, local non-crystallographic symmetry (NCS) restraints were employed in the refinement for better results. The four refined Fab models differ from one another by root-mean-square deviations (RMSD) of 0.21 Å – 0.35 Å for 381–413 Cα pairs, suggesting minimal variation in the polypeptide conformation. The two pairs of Fab (H/L, I/M and J/N, K/O) can be superposed by an RMSD of 0.53 Å between 851 matched pairs of Cα atoms. In either pair of the Fab an S-shaped PEG molecule was found at the pseudo-dyad. It binds to the N-terminal side of the Fab, which corresponds to the complementarity determining regions (CDRs), in a symmetrical way.

**Fig. 2:**
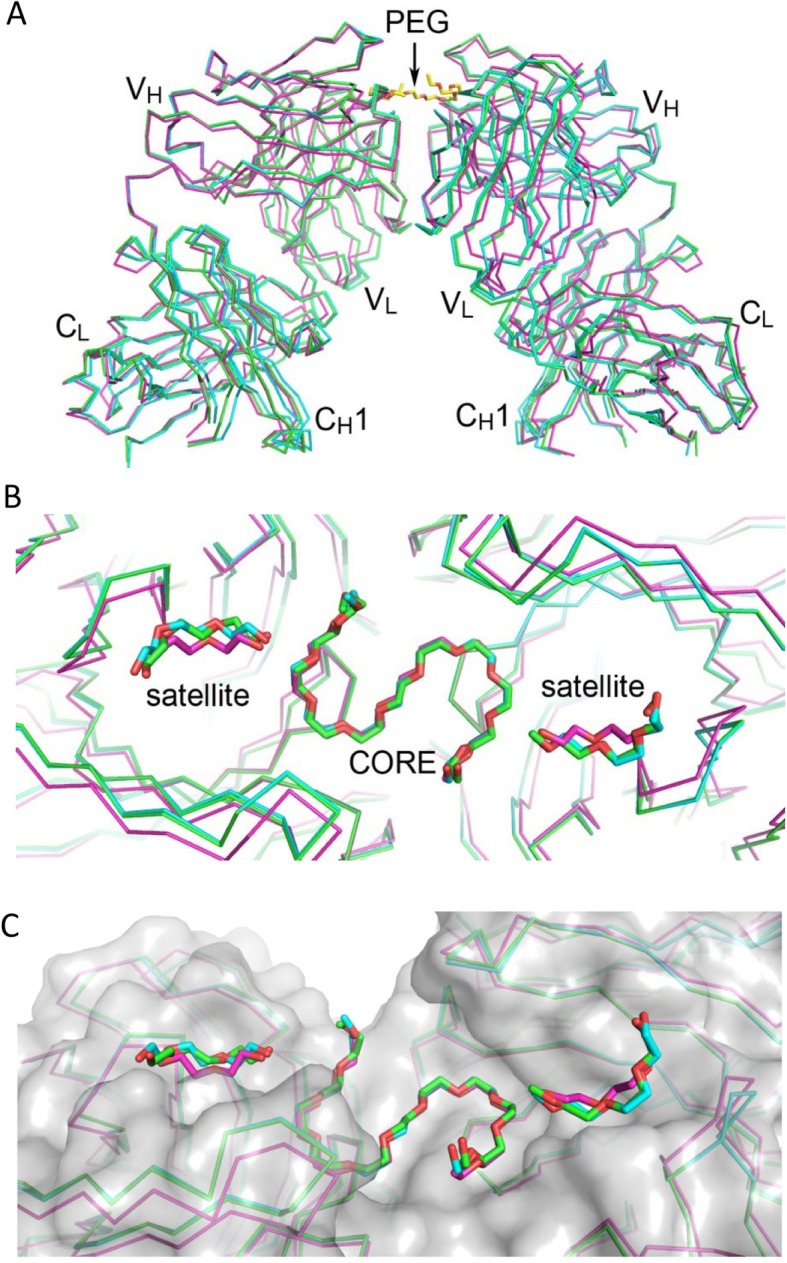
Fab dimer formation and PEG binding. **a** The two dimers of 3.3 Fab, colored in green and cyan, are superimposed on that of 2B5 Fab, in magenta, and all are shown as Cα-tracing diagrams. On top of the Fab dimers, the core fragment of PEG from the 2B5 Fab complex crystal is depicted as a yellow stick model, which is centered at the dyad axis. Locations of the V_H_, C_H_1, V_L_ and C_L_ domains in the Fab dimer are also indicated. **b** The bound PEG molecules are shown as stick models and colored according to their associated Fab dimers. The view is along the dyad axis of the Fab dimer, approximately from the top of (**a**). The core PEG fragment superimposes better than the satellite fragments. **c** The two molecules of 2B5 Fab are rendered with a translucent gray surface. The view is slightly tilted to show the topology of the PEG-binding regions

The tetragonal crystal of the 2B5-Fab/PEG complex contains only one Fab in its asymmetric unit (Fig. 1c). Nevertheless, a similar pair of Fab with an S-shaped PEG wedged between them can be generated by crystallographic 2-fold symmetry operation. This pair of Fab superimposes well on those of the monoclinic 3.3-Fab/PEG crystal by RMSD of 1.35 Å and 1.46 Å for 831 and 836 matched pairs of Cα atoms (Fig. 2a). The 2B5-Fab model shows RMSD of 0.38 Å – 0.46 Å for 355–382 Cα pairs from the four 3.3-Fab models, again suggesting a virtually identical polypeptide conformation.

In comparison, the refined model of 32D6-Fab in the trigonal crystal shows much larger conformational difference from those of 3.3-Fab and 2B5-Fab (Additional file 1: Figure S1), with RMSD of 2.28 Å – 2.48 Å for 356–366 matched pairs of Cα atoms. It contains two bound PEG molecules (Fig. 1d). One is bound to the N-terminal side but shows a different disposition on the protein surface with a different conformation from those in the 3.3-Fab and 2B5-Fab crystals. The other is bound to the central cavity of 32D6-Fab, far away from the CDRs.

Because 32D6 was expressed as a Fab and purified by His-affinity chromatography, whereas 3.3 and 2B5 were expressed as whole antibodies and the Fabs were obtained through papain digestion followed by PEG-affinity chromatography under acidic conditions, the different procedures employed to purify the Fabs might influence the subsequent experimental results. However, all three Fabs were successfully crystallized and their structures analyzed by X-ray diffraction. The structural analysis revealed a conserved specific binding mode of PEG to 3.3 and 2B5, which is different in many aspects from the non-specific binding modes to 32D6. Further analyses and comparisons are described in the following.

### Specific binding modes of PEG

As shown above, both 3.3-Fab and 2B5-Fab bind to PEG in a pairwise manner. In addition to the central S-shaped core fragment of PEG, there is a smaller satellite PEG fragment on each side of the core. Despite the different sizes of PEG (2000/4000 for 3.3-Fab and 8000 for 2B5-Fab) employed in crystallization, the Fab-bound PEG molecules observed in the crystals superimpose well (Fig. 2b). Between the core PEG fragments in the 3.3-Fab and 2B5-Fab complexes, the RMSD for the 34 and 32 equivalent non-hydrogen atoms vary slightly from 0.36 Å to 0.59 Å. The satellite fragments are also bound consistently to the same site in these two complex crystals of Fab/PEG. The sizes of the modeled PEG fragments, about 500 for the core and 200 for the satellite, are significantly smaller than those used in crystallization. Judging by their close proximity, it is likely that the bound core and satellite fragments to a Fab dimer were connected. In other words, they might belong to a single PEG molecule. The flexible regions between them were exposed to solvent and thus not visible. It is also possible that each fragment could belong to a different molecule of PEG. However, similar dispositions of the bound PEG fragments were observed regardless of the different sizes of PEG in the crystallization solutions, suggesting a conserved mode of specific binding.

**Fig. 3:**
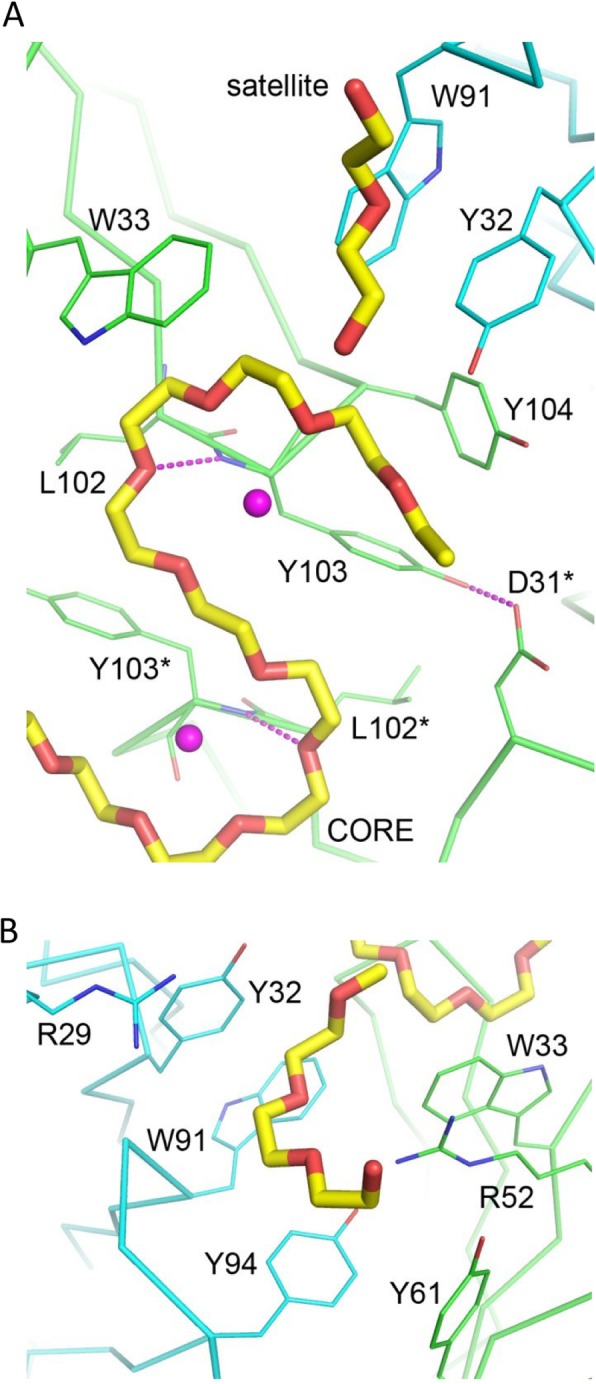
Specific Fab-PEG interactions. The PEG fragments are shown as thick stick models with yellow carbons. The protein models are shown as Cα-tracing diagrams. The heavy chains are colored green and the light chains cyan. The participating amino-acid side chains along with a few backbone parts are shown as thin sticks. The labels with asterisks denote the residues from the other monomer in a dimer. The two bound water molecules to the core PEG fragment are shown as pink spheres. Some hydrogen bonds are indicated by pink dashes. In (**a**) the view is centered at the core fragment. In (**b**) it is centered at the satellite fragment on the other side

The three central ethylene glycol units of the S-shaped PEG fits into a cleft at the Fab-Fab interface (Fig. 2c), flanked by the symmetry-related heavy-chain residues L102(H) and Y103(H) (Fig. 3a). The side chain of D31(H) at each end of the cleft forms a hydrogen bond to that of Y103(H) from the other Fab molecule. Here the PEG molecule takes a bend and starts to form a circular crown-ether-like structure. The next oxygen atom turns to make a hydrogen bond with the backbone nitrogen of Y103(H), and the two succeeding ethylene glycol units are stacked against the large planar indole group of the W33(H) side chain. Notably, at the center of each half-circle of the S-shaped PEG molecule, a water molecule is consistently observed, with distances of 2.6 Å – 3.4 Å from the four ether oxygen atoms that are directed toward it, making alternate hydrogen bonds that are reminiscent of those to the amino group of a “crowned” lysine side chain [23]. Beyond the fourth oxygen, additional packing interactions between the ethylene group and the side chain of Y104(H) are seen. Although the core PEG fragment is bound mostly by the heavy chain, it is also in slight contact with the side chains of Y32(L) and W91(L).

Despite the varying lengths, all satellite PEG fragments are bent with a crescent shape (Fig. 2c). Each is embedded in an open pocket formed by the side chains of W33(H), R52(H), Y61(H), R29(L), Y32(L), W91(L) and Y94(L), as shown in Fig. 3b. Both the heavy chain and the light chain contribute to the binding. Every satellite PEG fragment has its central part clamped between the guanidino groups of the extended side chains of R52(H) and R29(L) that approach each other from opposite directions. The remaining parts are packed against the planar side-chain groups of the aromatic amino acid residues. Interestingly, by assuming a similar conformation for the missing part between the S-shaped core fragment and the crescent-shaped satellite fragment, an unbroken model of PEG can be constructed (Additional file 1: Figure S2). Presumably the binding of a large PEG molecule to the Fab (either 3.3 or 2B5) would cover both the primary binding region with the core fragment and the secondary binding region with the satellite fragment.

### Other binding modes of PEG and crown ether

**Fig. 4:**
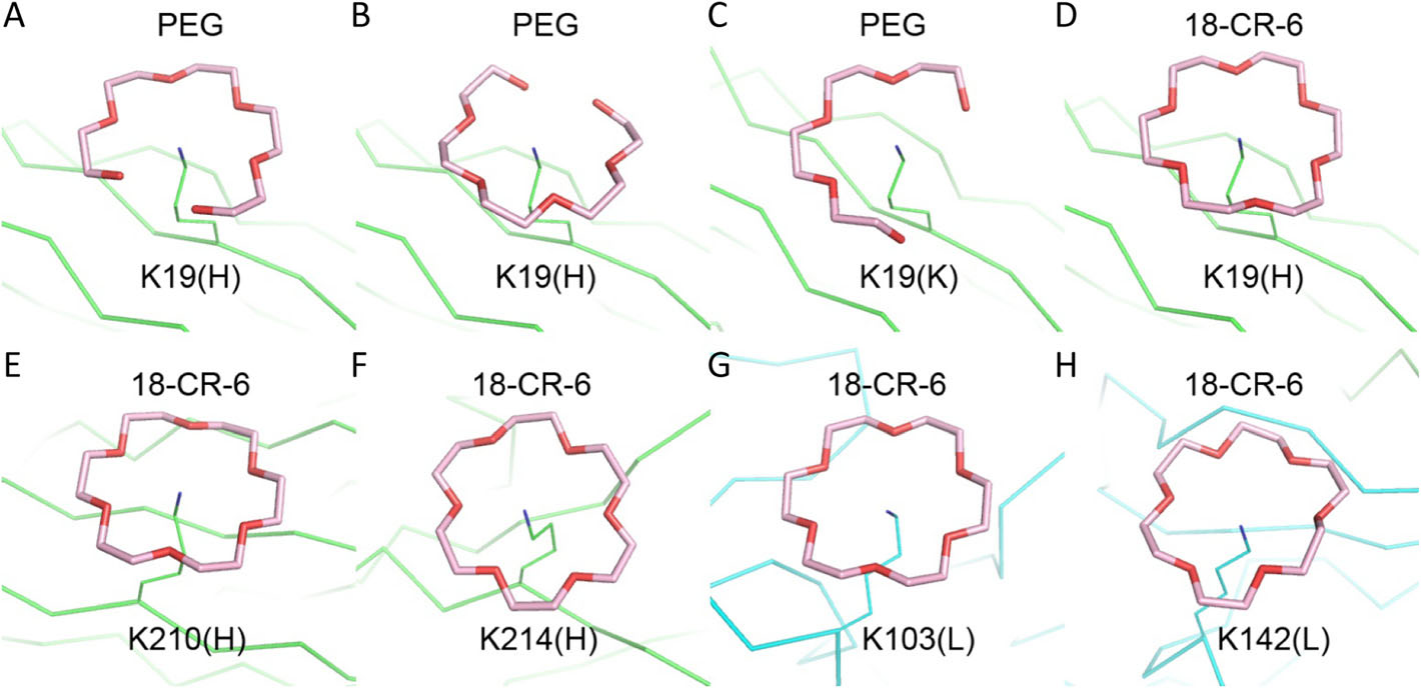
Binding of PEG and crown ether to lysine. The PEG fragments are shown as thick stick models in pink, and the lysine side chains as thin sticks in green and cyan for the heavy and light chains. The identity of ligands and the lysine residues are indicated on the top and at the bottom of each panel. In (**a**) and (**b**) the alternate binding modes to the same lysine side chain formed a closed ring structure, even though there was no crown ether in the 3.3-Fab crystal. In (**c**) the PEG fragment bound to the same lysine in another 3.3-Fab is more open. In (**d**) a real crown ether is bound to the equivalent lysine in the 2B5-Fab crystal. In (**e**) – (**h**) each lysine has crown ether bound in a similar mode

From the above observations, PEG molecules appear to be recognized by the antibodies 3.3 and 2B5 through a bent conformation that is similar to a portion of the circular structure of crown ether. In fact, although the 3.3-Fab/PEG complex crystal did not contain crown ether, some PEG fragments were bound to the side chains of K19(H) in a crown ether-like manner (Fig. 4a, b). Each had the PEG molecules disposed around the amino group of the lysine side chain, which assumed an outstretched conformation roughly perpendicular to the plane of PEG. In one place the circular electron densities strongly but falsely suggested the presence of crown ether, and were interpreted by alternative binding modes of a PEG fragment. In another place the U-shaped PEG is more similar in structure to one half of the S-shaped CDR-bound core fragment than the crescent-like satellite (Fig. 4c). The 2B5-Fab/PEG crystal contained real crown ether. In addition to the K19(H) side chain (Fig. 4d), crown ether was also observed to bind to those of K210(H), K214(H), K103(L) and K142(L) by a similar mode (Fig. 4e-h), as seen in previous crown ether-containing crystal structures [23].

**Fig. 5:**
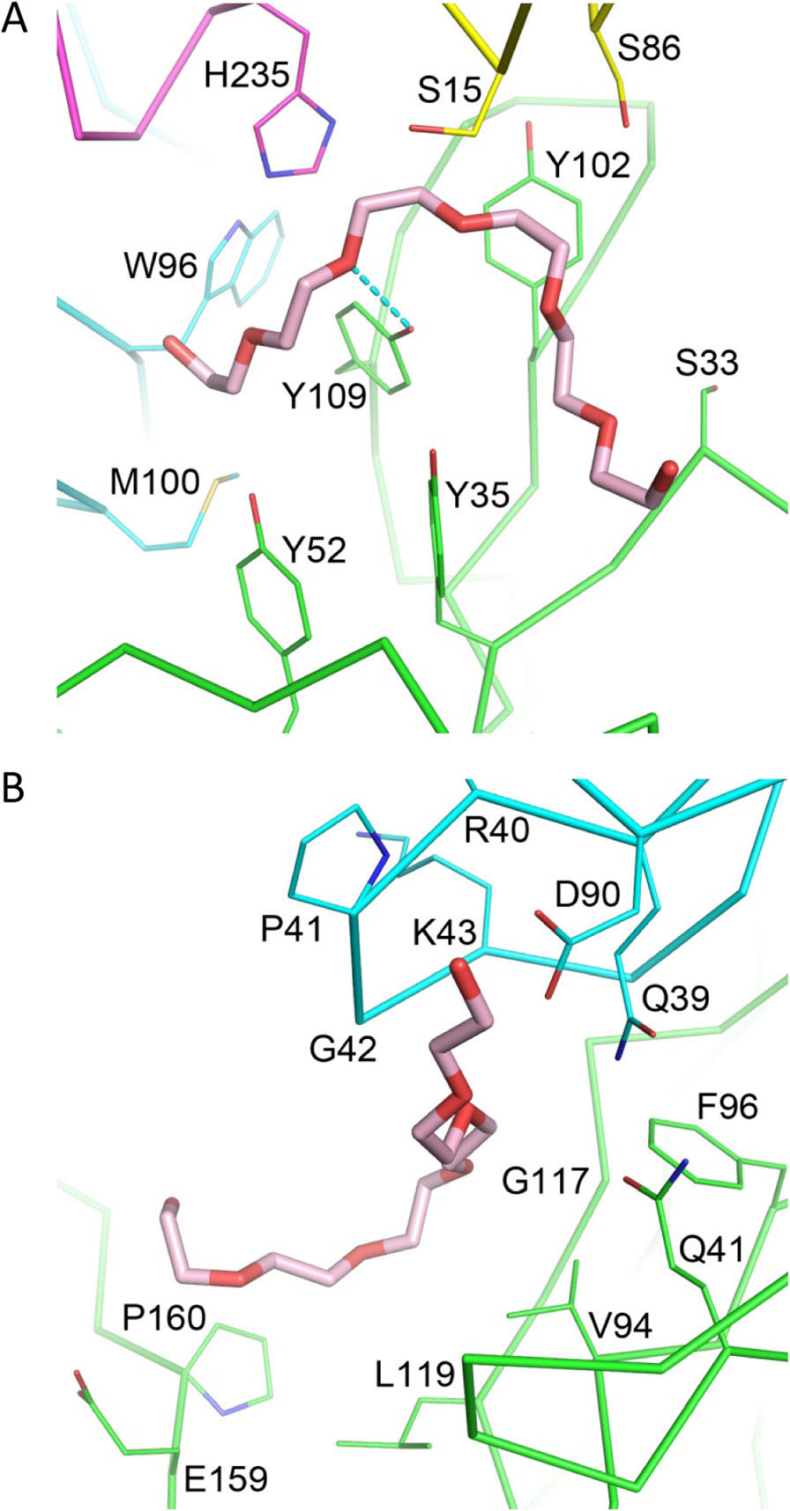
PEG binding in the 32D6-Fab crystal. **a** The first PEG fragment is shown as a thick stick model in pink. The protein molecules are shown as Cα-tracing diagrams, in green and cyan for the heavy and light chains of the primary Fab molecule, and in yellow and magenta for the heavy chains of two neighboring Fab related by crystallographic symmetry. Selected amino-acid side chains are shown as thin sticks. Potential hydrogen bonds are denoted by cyan dashes. **b** The second PEG fragment is shown in a similar way as in (**a**)

On the other hand, the N-terminally bound PEG molecule to the Fv region in the 32D6-Fab crystal adopts a rather extended conformation (Fig. 5a). It is much more exposed to the solvent, and makes contacts to three different Fab molecules related by crystallographic lattice symmetry. The PEG molecule interacts with the first Fab mainly via van der Waals contacts with at least five heavy-chain residues and two light-chain residues. The four aromatic side chains of Y35(H), Y52(H), Y102(H) and Y109(H) are most involved, with a possible hydrogen bond formation between the hydroxyl group of Y109(H) and an oxygen atom of the PEG. The PEG interacts with the other protein molecules through van der Waals contacts with the side chains of S15(H) and S86(H) from the second Fab, and with H235(H) from the third. All these interactions appear to be non-specific in nature.

The PEG molecule bound to the elbow region of 32D6-Fab also shows an extended conformation (Fig. 5b), which fits into a shallow groove at the interface of heavy chain and light chain. The residues that are in contact with the bound PEG include Q41(H), V94(H), F96(H), G117(H) and L119(H) from the V_H_ domain, E159(H) and P160(H) from the C_H_1 domain, and Q39(L), R40(L), P41(L), G42(L), K43(L) and D90(L) from the V_L_ domain. Both polar and non-polar interactions are likely to occur, but no specific hydrogen bond or hydrophobic patch could be identified.

### Formation of fab dimer by PEG binding

**Fig. 6:**
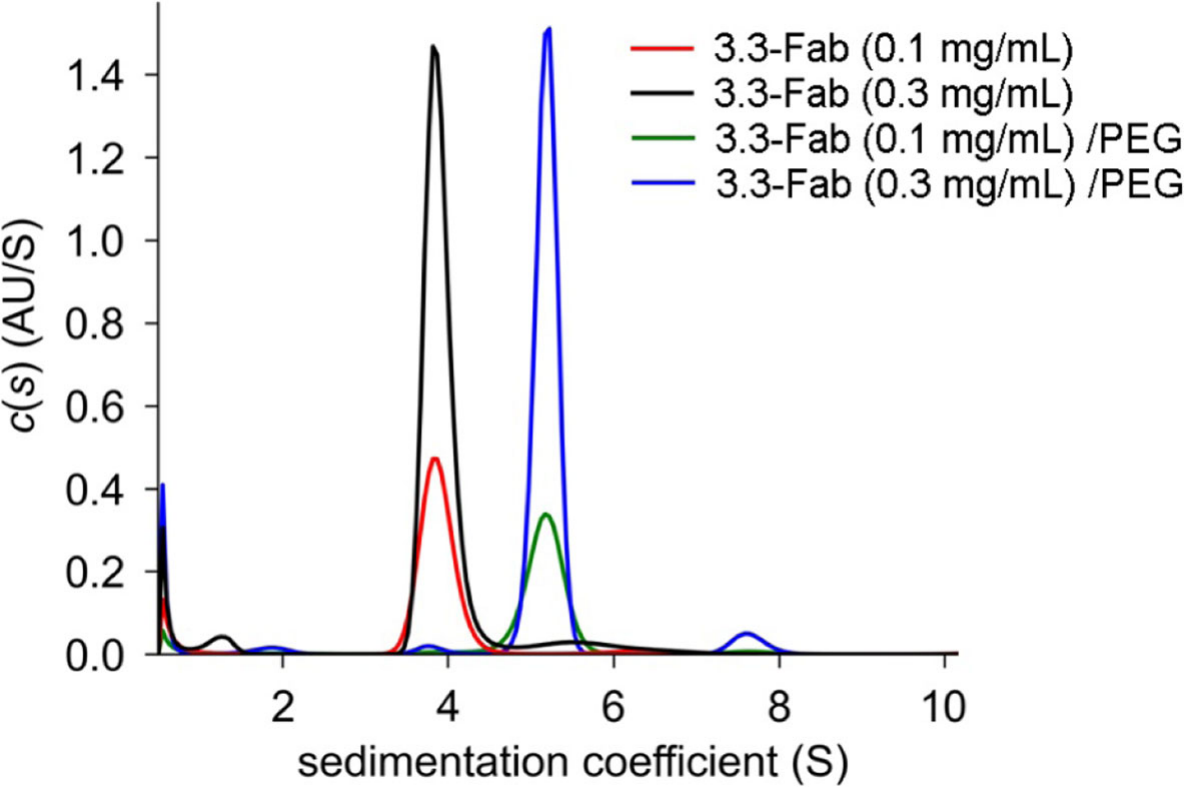
Dimer formation of 3.3-Fab in solution. The results of AUC are plotted here as curves of c(s) distribution against sedimentation coefficient. For 3.3-Fab alone the curve peaked at 3.863 S, corresponding to a molecular weight of 47.8 kDa with a friction ratio of 1.193. For 3.3-Fab and 1% PEG-550-MME it peaked at 5.167 S, suggesting a molecular weight of 90.2 kDa with a friction ratio of 1.392. These two sets of peaks indicate that the Fab formed a dimer in the presence of PEG, but existed as a monomer without PEG

The shared pairwise PEG-binding mode of Fab as observed in different crystal forms implicates physiological significance of the formation of Fab dimers. On each 3.3 Fab molecule, about 605 Å^2^ (or 3%) of the 19,600 Å^2^ surface area was buried by the PEG-mediated dimer formation. The heavy chain contributes 445 Å^2^ surface area, with at least 10 amino acid residues involved, and the light chain contributes 160 Å^2^, involving at least 6 residues. The 2B5 Fab has about 575 Å^2^ surface area buried, 405 Å^2^ on the heavy chain and 170 Å^2^ on the light chain, involving more than 9 and 6 residues, respectively. Although in both cases the Fab-Fab interface is significantly smaller than most other protein-protein interaction (PPI) areas that range from 1200 Å^2^ to 2000 Å^2^, it is comparable to those in the active-site-like PPI for transient docking [24, 25]. Presumably the Fab dimers of 3.3 and 2B5 do not form spontaneously in the absence of PEG. In fact, the subsequent AUC experiments demonstrated that 3.3-Fab exists as a monomer with a size of about 48 kDa in PEG-free solution but forms a dimer of about 90 kDa in the presence of PEG (Fig. 6). Both curves for 0.1 and 0.3 mg/mL 3.3-Fab alone show that most of the Fab was in monomeric form. On the contrary, both curves for 0.1 and 0.3 mg/mL 3.3-Fab with the addition of PEG-550-MME indicate that most of the Fab was in dimeric form. The transition from monomer to dimer appears to be concentration independent, and is most likely a result of the presence of PEG in solution.

**Fig. 7:**
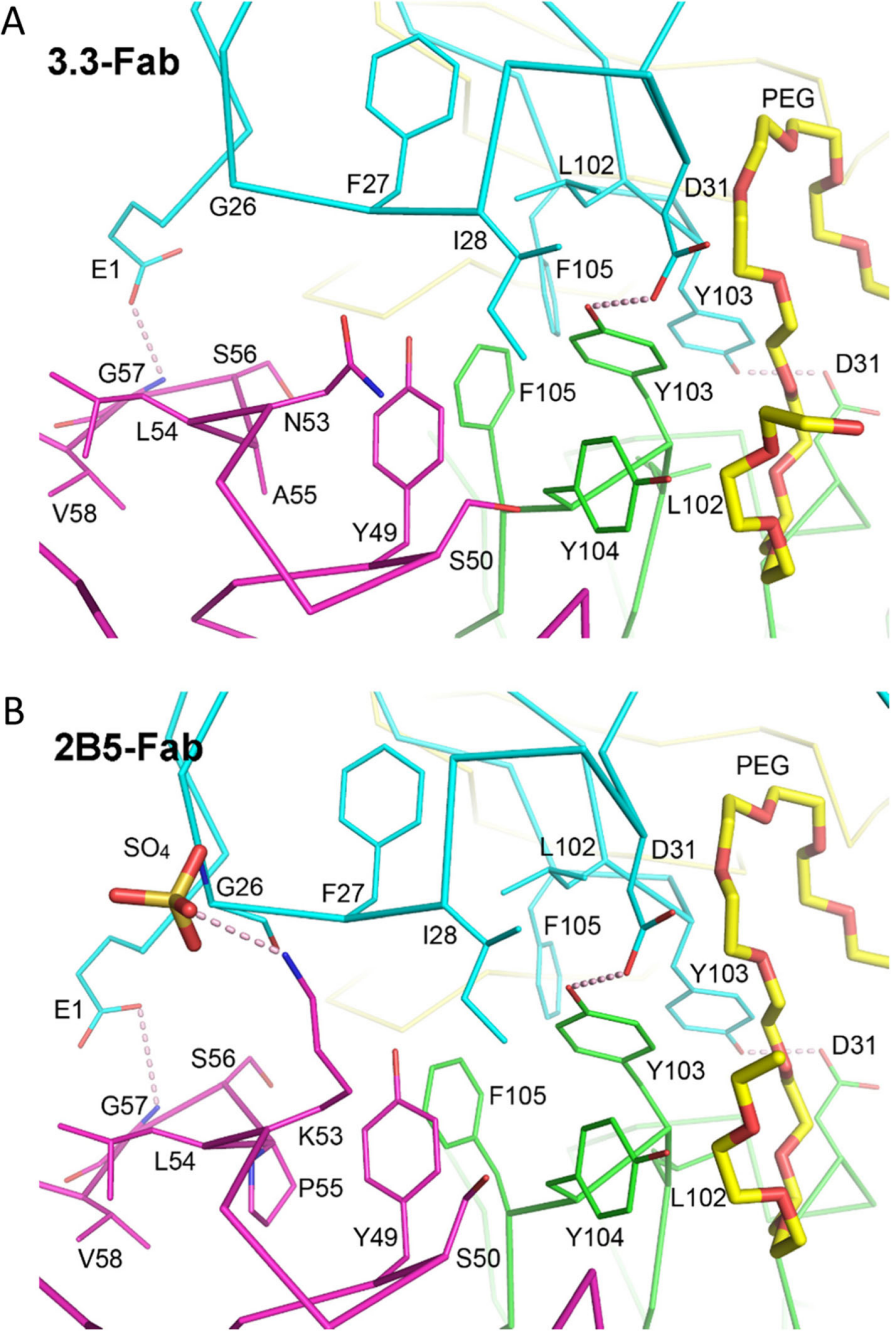
Dimer interface of PEG-bound Fab. **a** The first dimer from the 3.3-Fab/PEG crystal is shown as Cα-tracing diagrams, colored cyan and yellow for the heavy and light chains of one Fab, and green and magenta for another. The central PEG fragment is shown as yellow sticks, and the participating residues in the interface as thin sticks, colored according to the protein chains. Potential hydrogen bonds are indicated by pink dashes. **b** A dimer from the 2B5-Fab/PEG crystal is shown in a similar way as in (**a**). The sulfate ion, shown as an orange stick model, makes hydrogen bonds to the G26 backbone of one Fab and the K53 side chain of another

Participating amino acid residues in the PPI of the 3.3 Fab dimer are found at the N-terminus and in the loops G26 – D31 and L102 – F105 of the heavy chain, as well as in the loop Y49 – V58 of the light chain (Fig. 7a). In a free Fab E1(H) is highly exposed to the solvent, but in a dimer it is covered by S56(L), G57(L) and V58(L) from the other Fab, with a hydrogen bond formed between the carboxylate side chain of E1(H) and the backbone nitrogen of G57(L). The residues G26(H), F27(H) and I28(H) of one Fab are in contact with Y49(L), S50(L), L54(L), A55(L) and Y104(H) of another. The side chains of D31(H) and Y103(H) from two different Fab molecules also make a hydrogen bond, as described above in PEG binding. At the two-fold axis of the Fab dimer, L102(H) and Y103(H) packed against the symmetry-related equivalents to form the PEG-binding cleft, which is further buttressed by the two juxtaposed F105(H) side chains from the interior.

The PPI of the 2B5 Fab dimer is very similar to that of 3.3 Fab (Fig. 7b). Likewise, the three most involved residues are E1, I28 and Y103 in the heavy chain. Many interactions are conserved in the 2B5 and 3.3 Fab dimers, including the E1(H)-G57(L) and D31(H)-Y103(H) hydrogen bonds and the I28(H)-Y49(L)/Y104(H) contacts. However, in the light chain, K53 replaces L54 as the most involved residue. In the 2B5 Fab dimer, the side chain of K53(L) from one Fab is not only in contact with the backbone of F27(H) and the side chain of I28(H) but also bound to a sulfate ion from the crystallization solution that in turn is bound to the backbone of G26(H) from the second Fab. K53(L) in 2B5 corresponds to N53(L) in 3.3, and it is one of the three different amino acid residues in these two antibodies [13]. N53(L) makes few contacts with the other 3.3 Fab in a dimer, and there is no bound sulfate in the crystal structure. The second residue is the nearby P55(L) in 2B5, which replaces A55(L) in 3.3. However, as seen in the crystal structures, this variation does not result in significant conformation change. The third residue, V23(H) in 2B5 or A23(H) in 3.3, is facing the solvent and does not participate in the PPI or in PEG binding.

## Discussion

Owing to its simple chemical composition, PEG is generally considered biologically inert and safe for a wide range of applications, including medical use. Nevertheless, a small number of people have become allergic to it [26]. Presumably, the patients developed anti-PEG antibodies upon exposure to various PEG-containing products. Here by analyzing two crystal structures of anti-PEG Fab in complex with PEG, we demonstrate how an antibody specifically recognizes the antigen. Regardless of the different unit cells and space groups, the crystal structures of 3.3-Fab/PEG and 2B5-Fab/PEG complexes consistently show a conserved binding mode with two dyad-related molecules of Fab bound to the same S-shaped PEG core fragment in the center. The PEG binding site is formed mainly by the heavy chain, which contributes two CDR1 residues and three CDR3 residues. A nearby satellite PEG binding site suggests possible extension from the core fragment when the polymer chain is longer. This site is formed by both heavy chain and light chain, involving one CDR1 residue and two CDR2 residues from the former, and two CDR1 residues and two CDR3 residues from the latter. Remarkably, none of the three varied amino acid residues in 3.3-Fab/2B5-Fab, either A23/V23 in the heavy chain or N53/K53 and A55/P55 in the light chain, are involved in PEG binding. Therefore, other non-PEG interactions should be responsible for the previously reported temperature sensitivity of 2B5 [13].

Because two Fab molecules bind cooperatively to 1 S-shaped PEG, the PPI of the Fab dimer should be an important factor that determines the Fab-PEG affinity. Although dimer formation of 2B5-Fab buries slightly smaller surface areas than that of 3.3 Fab, additional sulfate-mediated interactions between K53(L) and G26(H) from two symmetry-related 2B5 Fab are likely to make it a stronger dimer. Under physiological conditions, the sulfate ions shall be replaced by phosphates, which are as capable of hydrogen bonding to the K53(L) side chain and the G26(H) backbone. On the other hand, higher temperatures can weaken the anion bonds, make the lysine side chain more flexible, and disrupt the phosphate-mediated PPI. Thus it becomes less favored to form the 2B5-Fab dimer when the temperature is raised. Consequently, 2B5 binds better to PEG than does 3.3 at lower temperatures such as 4 °C, but worse at higher temperatures such as 25 °C, as demonstrated in our previous work [13]. In fact, crystallization of the 2B5-Fab/PEG complex failed at 18 °C, and we had to use 10 °C.

In the previous work we also showed that crown ether (18-crown-6) can inhibit 2B5 binding to PEG in a dose dependent manner, while it was a poor inhibitor for 3.3 [13]. In that work it was proposed that 3.3 recognizes some existing PEG conformation at all temperatures, and 2B5 selectively binds to a crown ether-like structure at low temperature. It is true that PEG can assume a crown ether-like conformation especially at lower temperatures, but it is also true that the antibody binds to some crown ether-like PEG structure. Thus the observed temperature-dependent difference between 3.3 and 2B5 is unlikely a result of the PEG conformation. Not only was PEG seen to bind to the K19(H) side chain of 3.3-Fab in exactly a crown ether-like manner, but the core PEG fragment bound to either 3.3-Fab or 2B5-Fab was folded into the same S-shaped conformation that can also be represented by two dyad-related crown ether-like moieties. Instead, the inhibition effect of crown ether on 2B5 is more likely a result of its binding to K53(L), preventing dimer formation, rather than directly blocking the PEG binding site. Remarkably, 50 mM crown ether was present in the crystallization solution of the 2B5-Fab/PEG complex. The molar concentration of 30% PEG-8000 was slightly lower than that of crown ether, but it was still able to bind to the Fab. Higher temperatures (such as 18 °C) could nevertheless render PEG molecules in favor of extended conformations and reduce its affinity to the Fab. The flexible side chain of K53(L) may also favor an extended conformation that would easily become “crowned”, making the Fab dimer formation difficult.

Interestingly, the AUC results showed that PEG-550-MME was effective in crosslinking two 3.3-Fab molecules. The chain length PEG-550-MME is just sufficient to cover the S-shaped core PEG fragment in the center of the 3.3-Fab/PEG (or 2B5-Fab/PEG) complex structure. Therefore, the observed 3.3-Fab/PEG-550-MME complex was probably made up by two 3.3-Fab bound to a single molecule of PEG-550-MME. Although each satellite PEG binding site might contain an additional PEG molecule, because it is constituted by amino-acid residues from only one Fab molecule, its occupancy should not contribute to the Fab dimer formation. The synergic interactions of two Fab molecules with the S-shaped core PEG fragment are thus the primary determinant for the antigen recognition. The interactions with additional PEG moieties in the satellite binding site can enhance the binding strength, but only for a larger PEG molecule. On the other hand, the long PEG molecule on PEGylated protein drug may adopt the crown ether-like module when caught by protein surface lysine, and act like an envelope that blocks some protein surface region to reduce the immunogenicity.

Furthermore, we also investigated whether the two-fold relationship in a PEG-bound Fab dimer can be extended to an IgG molecule. By using the intact antibody structure of PDB 1IGT [27] as a template, a crude model of full-length 2B5 IgG was constructed in which the two-fold symmetry was preserved as a crystallographic dyad (Additional file 1: Figure S3A). This seems to be a plausible model, but the problem is a 70 Å distance between the two heavy-chain C-termini of the Fab. The connection to Fc would require large structural rearrangements in the hinge region that would break up the disulfide cross-links between the conserved cysteine residues. Consequently, the two Fab molecules which bind to the same S-shaped core PEG fragment are more likely to come from two separate antibodies (Additional file 1: Figure S3B). In a recent study, we showed that the required size of PEG for binding to the anti-PEG antibody 3.3 IgG should be more than 1000 Da but might be less than 2000 Da [28]. It is consistent with the combined length of a core PEG fragment and two satellite fragments (Additional file 1: Figure S3A), whose size would be about 1500 Da. An additional short fragment would be necessary for anchoring to the ELISA plate.

In this study, we determined the crystal structures of two PEG-specific Fab and one nonspecific Fab in complex with PEG. Both specific Fab/PEG complexes share a similar dimeric binding mode that is very different from that of the nonspecific complex. Antigen recognition by symmetrical binding of two Fab is rarely observed. An example is the Fab complex structure of antibody b12 against human immunodeficiency virus type 1 (HIV-1) with the peptide mimic B2.1 (PDB 1NOX), in which the antigen B2.1 is functional as a disulfide-bridged homodimer and each b12 Fab binds to one chain but not the other of B2.1 [29]. Unlike the Fab/PEG complex, in the b12/B2.1 complex the two Fab molecules do not interact with each other. Therefore, dimer formation of b12 Fab is likely a result of the dimeric nature of the antigen. Dimer formations without antigen have also been observed in therapeutic monoclonal antibodies [30]. They can be a result of domain exchange or other covalent and noncovalent interactions between the antibodies, probably induced by stress [30, 31]. In contrast, PEG binding to the CDR region of Fab is more common. For example, in the crystal structures of Fab against morphine and cocaine (PDB 1Q0X and 2AJS), PEG was found in the CDR region [32, 33]. Interestingly, in both structures the PEG molecules are bound to the Fab mainly through van der Waals contacts with aromatic side chains (Additional file 1: Figure S4). Similar interactions are seen in the 3.3-Fab/PEG and 2B5-Fab/PEG structures, as well as 32D6-Fab/PEG. Probably the frequent presence of aromatic side chains in the CDR region is inviting to PEG binding, either specifically or nonspecifically.

## Conclusions

Although many complex structures of Fab with other haptens have been reported, the structure presented here is the first specific complex with PEG, a simple but large “hapten” that comprises plain repetitive units. By analyzing the crystal structures, it is now clear how the antibodies 3.3 and 2B5 recognize PEG in a specific manner, although we need to mention here that other anti-PEG antibodies may bind to PEG by a different mechanism. In 3.3 and 2B5, CDR1 and CDR3 of two symmetry-related heavy chains constitute the binding site for the S-shaped core PEG fragment. The adjacent binding site for the satellite PEG fragment is formed by CDR1 and CDR2 of the heavy chain and CDR1 and CDR3 of the light chain. In addition to CDR1 and CDR3 of the heavy chain, the PPI between two Fab molecules in a dimer also involves CDR2 of the light chain. The important K53(L) is a CDR2 residue. Taken together, each of the three CDRs in both heavy chain and light chain plays an important role in the recognition of PEG as an antigen. Elucidation of the Fab-PEG interactions allows rational designs to improve the antibodies for better use. For example, protein engineering by mutating K53(L) of 2B5 to arginine could enhance the affinity and/or reduce the temperature sensitivity. The positively charged planar side chain of R53(L) would stack better with the backbone atoms of F27(H) but maintain the original hydrogen bond and possibly make another one to the phosphate ion.

## Supplementary information

**Additional file 1:** **Table S1.** Dissociation constants K_D_ of PEG and antibodies 3.3 and 2B5. **Table S2.** Sequence comparison in the Fv regions of 3.3 and 2B5. **Figure S1.** Comparison of the 32D6, 3.3 and 2B5 Fab structures. The superimposed Fab structures are colored in orange, blue and green, respectively. **Figure S2.** Extension of PEG molecule. **Figure S3.** Full-length IgG/PEG models. **Figure S4.** Two other examples of non-specific PEG-Fab complex structures.

## Data Availability

Data and materials are available from the corresponding author on reasonable request.

## References

[1] Harris JM, Chess RB (2003). Effect of pegylation on pharmaceuticals. Nat Rev Drug Discov.

[2] Kim S, Lim YT, Soltesz EG, De Grand AM, Lee J, Nakayama A, Parker JA, Mihaljevic T, Laurence RG, Dor DM, Cohn LH, Bawendi MG, Frangioni JV (2004). Near-infrared fluorescent type II quantum dots for sentinel lymph node mapping. Nat Biotechnol.

[3] Lien MY, Liu LC, Wang HC, Yeh MH, Chen CJ, Yeh SP, Bai LY, Liao YM, Lin CY, Hsieh CY, Lin CC, Li LY, Lin PH, Chiu CF (2014). Safety and efficacy of pegylated liposomal doxorubicin-based adjuvant chemotherapy in patients with stage I-III triple-negative breast cancer. Anticancer Res.

[4] Manns MP, McHutchison JG, Gordon SC, Rustgi VK, Shiffman M, Reindollar R, Goodman ZD, Koury K, Ling M, Albrecht JK (2001). Peginterferon alfa-2b plus ribavirin compared with interferon alfa-2b plus ribavirin for initial treatment of chronic hepatitis C: a randomised trial. Lancet..

[5] Schreiber S, Khaliq-Kareemi M, Lawrance IC, Thomsen OØ, Hanauer SB, McColm J, Bloomfield R, Sandborn WJ (2007). Maintenance therapy with certolizumab pegol for Crohn's disease. N Engl J Med.

[6] Cheng TC, Chuang KH, Chen M, Wang HE, Tzou SC, Su YC, Chuang CH, Kao CH, Chen BM, Chang LS, Roffler SR, Cheng TL (2013). Sensitivity of PEGylated interferon detection by anti-polyethylene glycol (PEG) antibodies depends on PEG length. Bioconjug Chem.

[7] Su YC, Chen BM, Chuang KH, Cheng TL, Roffler SR (2010). Sensitive quantification of PEGylated compounds by second-generation anti-poly (ethylene glycol) monoclonal antibodies. Bioconjug Chem.

[8] Kao CH, Wang JY, Chuang KH, Chuang CH, Cheng TC, Hsieh YC, Tseng YL, Chen BM, Roffler SR, Cheng TL (2014). One-step mixing with humanized anti-mPEG bispecific antibody enhances tumor accumulation and therapeutic efficacy of mPEGylated nanoparticles. Biomaterials..

[9] Su YC, Burnouf PA, Chuang KH, Chen BM, Cheng TL, Roffler SR (2017). Conditional internalization of PEGylated nanomedicines by PEG engagers for triple negative breast cancer therapy. Nat Commun.

[10] Tung HY, Su YC, Chen BM, Burnouf PA, Huang WC, Chuang KH, Yan YT, Cheng TL, Roffler SR (2015). Selective delivery of PEGylated compounds to tumor cells by anti-PEG hybrid antibodies. Mol Cancer Ther.

[11] Cheng TL, Wu PY, Wu MF, Chern JW, Roffler SR (1999). Accelerated clearance of polyethylene glycol-modified proteins by anti-polyethylene glycol IgM. Bioconjug Chem.

[12] Cheng TL, Cheng CM, Chen BM, Tsao DA, Chuang KH, Hsiao SW, Lin YH, Roffler SR (2005). Monoclonal antibody-based quantitation of poly (ethylene glycol)-derivatized proteins, liposomes, and nanoparticles. Bioconjug Chem.

[13] Su YC, Al-Qaisi TS, Tung HY, Cheng TL, Chuang KH, Chen BM, Roffler SR (2014). Mimicking the germinal center reaction in hybridoma cells to isolate temperature-selective anti-PEG antibodies. MAbs.

[14] Lee CC, Yang CY, Lin LL, Ko TP, Chang AH, Chang SS, Wang AH (2019). An effective neutralizing antibody against influenza virus H1N1 from human B cells. Sci Rep.

[15] Otwinowski Z, Minor W (1997). Processing of X-ray diffraction data collected in oscillation mode. Methods Enzymol.

[16] Winn MD, Ballard CC, Cowtan KD, Dodson EJ, Emsley P, Evans PR, Keegan RM, Krissinel EB, Leslie AG, McCoy A, McNicholas SJ, Murshudov GN, Pannu NS, Potterton EA, Powell HR, Read RJ, Vagin A, Wilson KS (2011). Overview of the CCP4 suite and current developments. Acta Crystallogr D Biol Crystallogr.

[17] Lee CC, Lin LL, Chan WE, Ko TP, Lai JS, Wang AH (2013). Structural basis for the antibody neutralization of herpes simplex virus. Acta Crystallogr D Biol Crystallogr.

[18] Adams PD, Afonine PV, Bunkóczi G, Chen VB, Davis IW, Echols N, Headd JJ, Hung LW, Kapral GJ, Grosse-Kunstleve RW, McCoy AJ, Moriarty NW, Oeffner R, Read RJ, Richardson DC, Richardson JS, Terwilliger TC, Zwart PH (2010). PHENIX: a comprehensive python-based system for macromolecular structure solution. Acta Crystallogr D Biol Crystallogr.

[19] Emsley P, Lohkamp B, Scott WG, Cowtan K (2010). Features and development of Coot. Acta Crystallogr D Biol Crystallogr.

[20] Williams CJ, Headd JJ, Moriarty NW, Prisant MG, Videau LL, Deis LN, Verma V, Keedy DA, Hintze BJ, Chen VB, Jain S, Lewis SM, Arendall WB, Snoeyink J, Adams PD, Lovell SC, Richardson JS, Richardson DC (2018). MolProbity: more and better reference data for improved all-atom structure validation. Protein Sci.

[21] The PyMOL Molecular Graphics System, Version 2.0 Schrödinger, LLC.

[22] Pettersen EF, Goddard TD, Huang CC, Couch GS, Greenblatt DM, Meng EC, Ferrin TE (2004). UCSF chimera—a visualization system for exploratory research and analysis. J Comput Chem.

[23] Lee CC, Maestre-Reyna M, Hsu KC, Wang HC, Liu CI, Jeng WY, Lin LL, Wood R, Chou CC, Yang JM, Wang AH (2014). Crowning proteins: modulating the protein surface properties using crown ethers. Angew Chem Int Ed Eng.

[24] Ran X, Gestwicki JE (2018). Inhibitors of protein-protein interactions (PPIs): an analysis of scaffold choices and buried surface area. Curr Opin Chem Biol.

[25] Sable R, Jois S (2015). Surfing the protein-protein interaction surface using docking methods: application to the design of PPI inhibitors. Molecules.

[26] Wenande E, Garvey LH (2016). Immediate-type hypersensitivity to polyethylene glycols: a review. Clin Exp Allergy.

[27] Harris LJ, Larson SB, Hasel KW, Day J, Greenwood A, McPherson A (1992). The three-dimensional structure of an intact monoclonal antibody for canine lymphoma. Nature..

[28] Lin WW, Hsieh YC, Cheng YA, Chuang KH, Huang CC, Chuang CH, Chen IJ, Cheng KW, Lu YC, Cheng TC, Wang YT, Roffler SR, Cheng TL (2016). Optimization of an anti-poly (ethylene glycol) (anti-PEG) cell-based capture system to quantify PEG and PEGylated molecules. Anal Chem.

[29] Saphire EO, Montero M, Menendez A, van Houten NE, Irving MB, Pantophlet R, Zwick MB, Parren PW, Burton DR, Scott JK, Wilson IA (2007). Structure of a high-affinity "mimotope" peptide bound to HIV-1-neutralizing antibody b12 explains its inability to elicit gp120 cross-reactive antibodies. J Mol Biol.

[30] Plath F, Ringler P, Graff-Meyer A, Stahlberg H, Lauer ME, Rufer AC, Graewert MA, Svergun D, Gellermann G, Finkler C, Stracke JO, Koulov A, Schnaible V (2016). Characterization of mAb dimers reveals predominant dimer forms common in therapeutic mAbs. MAbs..

[31] Luo Y, Raso SW, Gallant J, Steinmeyer C, Mabuchi Y, Lu Z, Entrican C, Rouse JC (2017). Evidence for intermolecular domain exchange in the fab domains of dimer and oligomers of an IgG1 monoclonal antibody. MAbs..

[32] Pozharski E, Wilson MA, Hewagama A, Shanafelt AB, Petsko G, Ringe D (2004). Anchoring a cationic ligand: the structure of the fab fragment of the anti-morphine antibody 9B1 and its complex with morphine. J Mol Biol.

[33] Zhu X, Dickerson TJ, Rogers CJ, Kaufmann GF, Mee JM, McKenzie KM, Janda KD, Wilson IA (2006). Complete reaction cycle of a cocaine catalytic antibody at atomic resolution. Structure.

