# Supplementary material for: Structural basis of polyethylene glycol recognition by antibody
Source: J Biomed Sci. 2020 Jan 7;27:12. doi: 10.1186/s12929-019-0589-7 (PMC6945545; doi:10.1186/s12929-019-0589-7)

**Structural basis of polyethylene glycol recognition by antibody**

Cheng-Chung Lee^1$^*, Yu-Cheng Su^2$^, Tzu-Ping Ko^1$^, Li-Ling Lin^1^, Chih-Ya Yang^3^, Stanley Shi-Chung Chang^3,4^, Steve R. Roffler^5^*, Andrew H.-J. Wang^1^*

^1^Institute of Biological Chemistry, Academia Sinica, Taipei, Taiwan, ^2^Department of Biological Science and Technology, National Chiao Tung University, Hsin-Chu, Taiwan, ^3^Medigen Biotechnology Corporation, Taipei, Taiwan, ^4^Institute of Biotechnology, National Taiwan University, Taipei, Taiwan, ^5^Institute of Biomedical Sciences, Academia Sinica, Taipei, Taiwan.

$ CCL, YCS & TPK contributed equally to this work.

* Corresponding authors: CCL, [chengung@gate.sinica.edu.tw](mailto:chengung@gate.sinica.edu.tw); SRR, sroff@ibms.sinica.edu.tw; AHJW, [ahjwang@gate.sinica.edu.tw](mailto:ahjwang@gate.sinica.edu.tw)

**Supporting information**

Table S1. Dissociation constants K_D_ of PEG and antibodies 3.3 and 2B5.

|  | 4°C | 25°C | 37°C |
| --- | --- | --- | --- |
| 3.3 | (5.2 ± 0.50) X 10^-7^ M | (1.3 ± 0.17) X 10^-7^ M | (2.0 ± 0.11) X 10^-7^ M |
| 2B5 | (4.7 ± 0.06) X 10^-7^ M | (8.7 ± 0.47) X 10^-5^ M | (not measured) |

Table S2. Sequence comparison in the Fv regions of 3.3 and 2B5.

| **V_H_** | **//---------------FR1-----------------//** |
| --- | --- |
| **3.3** | **// G L V Q P G G S M K L S C A A S G //** |
| **2B5** | **// G L V Q P G G S M K L S C V A S G //** |
| **Residue number** | **(23)** |

| **V_L_** | **//-----CDR2------//------CDR3-------//** |
| --- | --- |
| **3.3** | **// S T S N L A S // Q W S S Y P R T //** |
| **2B5** | **// S T S K L P S // Q W S S Y P R T //** |
| **Residue number** | **(53) (55)** |

Figure S1. Comparison of the 32D6, 3.3 and 2B5 Fab structures. The superimposed Fab structures are colored in orange, blue and green, respectively.


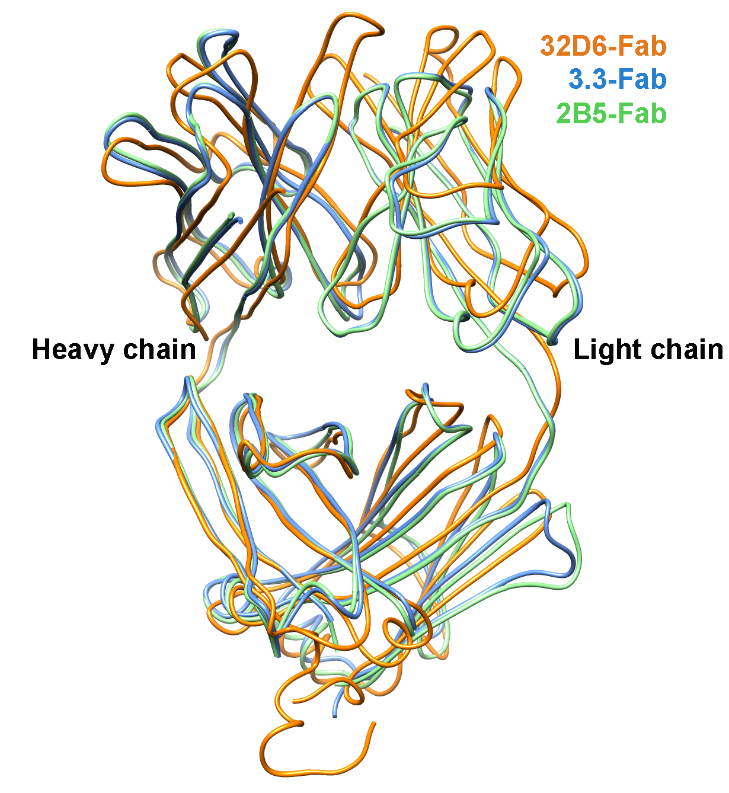


Figure S2. Extension of PEG molecule. The protein parts are shown as Cα-tracing diagrams and the bound PEG as stick models, both colored green/cyan for 3.3-Fab and yellow for 2B5. In (A) the PEG models were connected by using modules with a similar S-shaped conformation as that of the central core fragment. The result is shown here as a line model in pink. In (B) further extension of the PEG molecule was made by repetitive use of the same motif. The outcome is a large PEG molecule with a spiral conformation, shown here as a pink stick model.

A


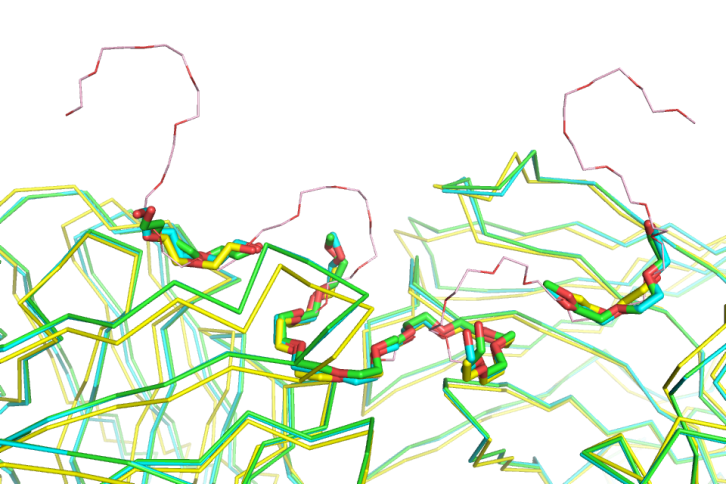


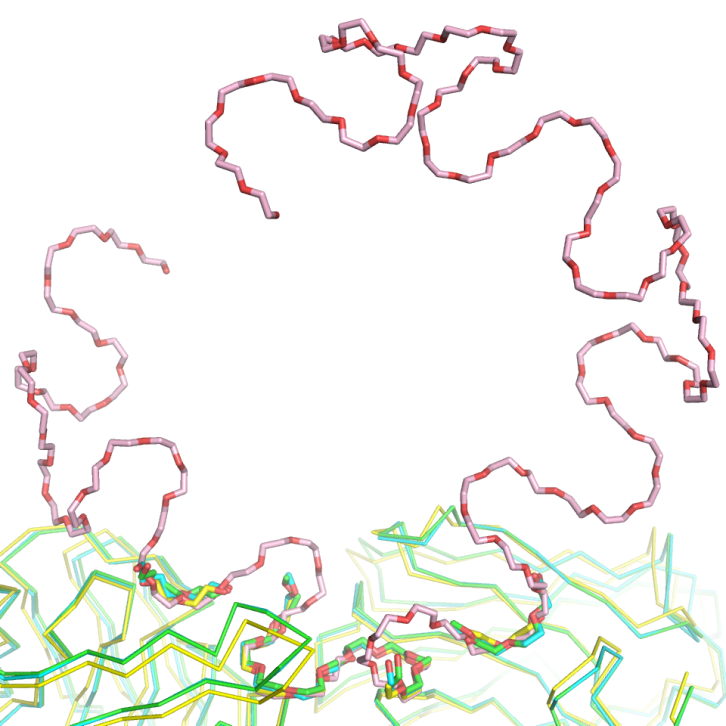


B

Figure S3. Full-length IgG/PEG models. IgG molecules are depicted as surface diagrams with the heavy chains colored cyan and gray. The light chains are in orange and pink. The PEG models are shown as worms with carbon and oxygen atoms colored green and red. (A) By assuming that the crystallographic dyad relationship between the Fab and Fc is conserved, a dimeric Fc was constructed based on the model from PDB 1IGT. However, it was necessary to relocate the linker between Fab and Fc, which are barely connected by the limited numbers of amino acid residues, disrupting the disulfide connections in the hinge region. (B) The two Fab molecules in a PEG-bound dimer were considered as belonging to two separate IgG molecules. Two molecules of IgG from PDB 1IGT were thus superimposed directly onto the Fab. The resulting model does not show any undesired contacts between the two IgG molecules, of which the hinge regions remain intact.


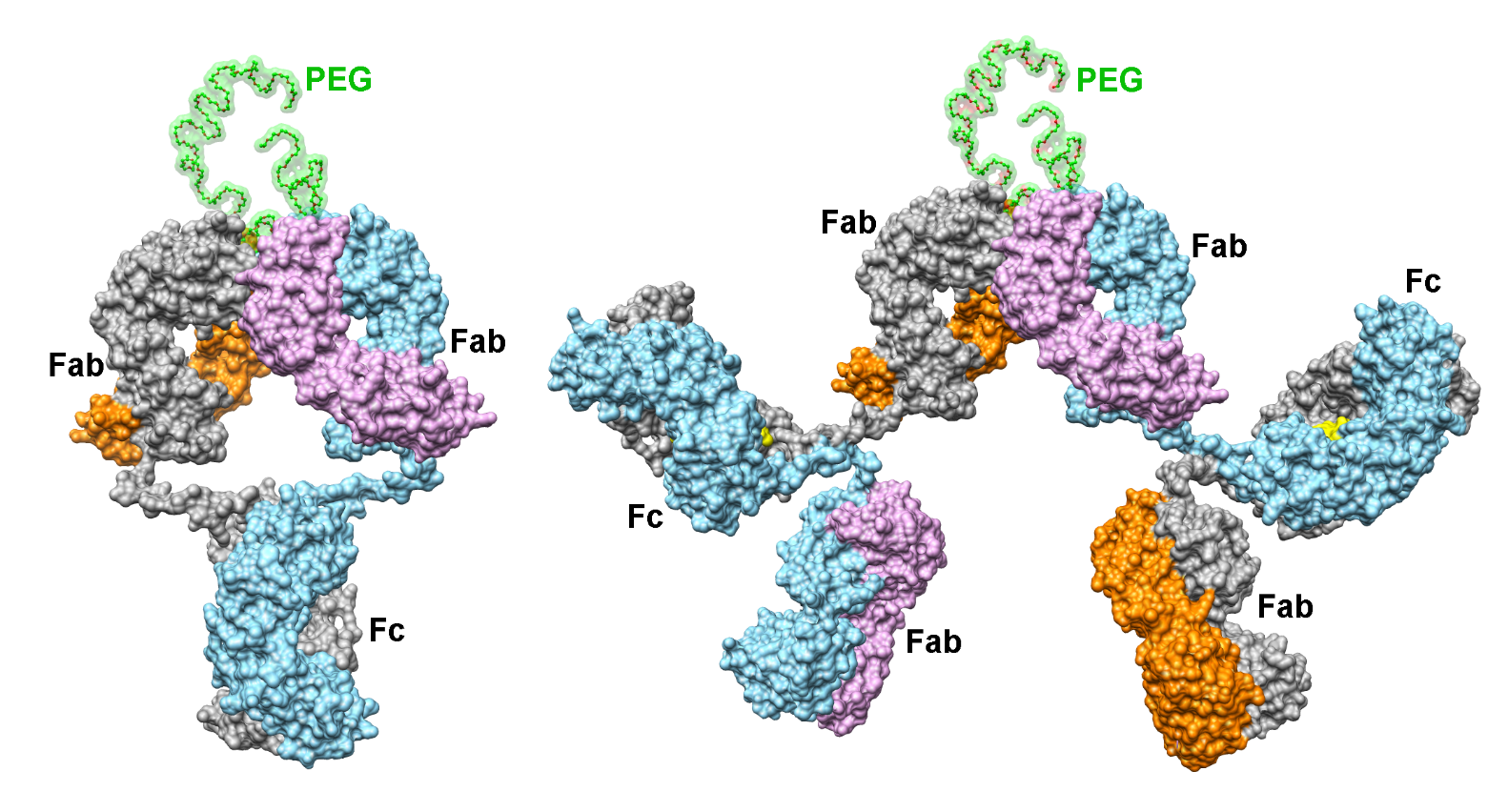


B

A

Figure S4. Two other examples of non-specific PEG-Fab complex structures.


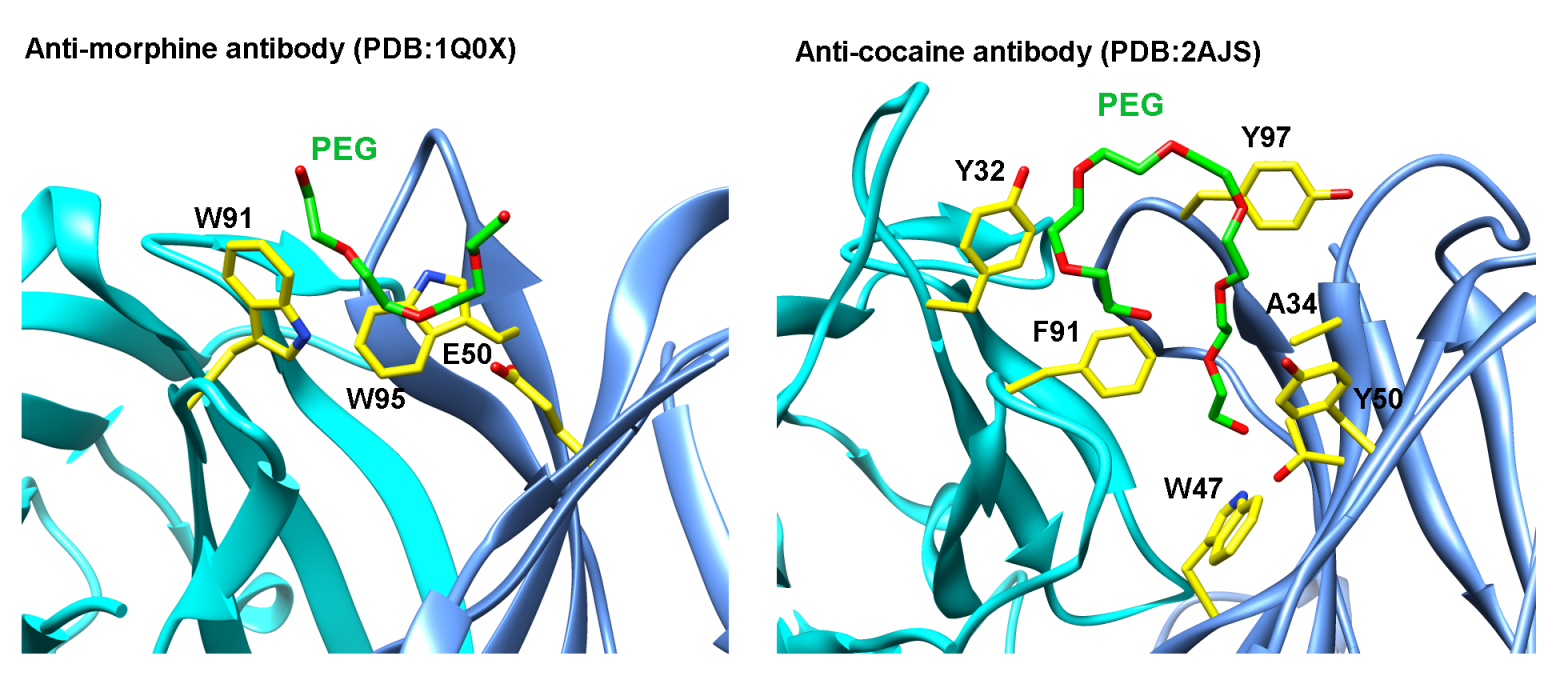

Supplement: Supplementary file 1 — Additional file 1: Table S1. Dissociation constants KD of PEG and antibodies 3.3 and 2B5. Table S2. Sequence comparison in the Fv regions of 3.3 and 2B5. Figure S1. Comparison of the 32D6, 3.3 and 2B5 Fab structures. The superimposed Fab structures are colored in orange, blue and green, respectively. Figure S2. Extension of PEG molecule. Figure S3. Full-length IgG/PEG models. Figure S4. Two other examples of non-specific PEG-Fab complex structures. [file 12929_2019_589_MOESM1_ESM.docx]
